# Effect of Mass Treatment with Azithromycin on Causes of Death in Children in Malawi: Secondary Analysis from the MORDOR Trial

**DOI:** 10.4269/ajtmh.19-0613

**Published:** 2020-04-27

**Authors:** John D. Hart, Khumbo Kalua, Jeremy D. Keenan, Thomas M. Lietman, Robin L. Bailey

**Affiliations:** 1 London School of Hygiene and Tropical Medicine, London, United Kingdom;; 2 Blantyre Institute for Community Outreach and College of Medicine, University of Malawi, Blantyre, Malawi;; 3 Department of Ophthalmology, Francis I. Proctor Foundation, University of California, San Francisco, San Francisco, California

## Abstract

Recent evidence indicates mass drug administration with azithromycin may reduce child mortality. This study uses verbal autopsy (VA) to investigate the causes of individual deaths during the Macrolides Oraux pour Réduire les Décès avec un Oeil sur la Résistance (MORDOR) trial in Malawi. Cluster randomization was performed as part of MORDOR. Biannual household visits were conducted to distribute azithromycin or placebo to children aged 1–59 months and update the census to identify deaths for VA. MORDOR was not powered to investigate mortality effects at individual sites, but the available evidence is presented here for hypothesis generation regarding the mechanism through which azithromycin may reduce child mortality. Automated VA analysis was performed to infer the likely cause of death using two major analysis programs, InterVA and SmartVA. A total of 334 communities were randomized to azithromycin or placebo, with more than 130,000 person-years of follow-up. During the study, there were 1,184 deaths, of which 1,131 were followed up with VA. Mortality was 9% lower in azithromycin-treated communities than in placebo communities (rate ratio 0.91 [95% CI: 0.79–1.05]; *P* = 0.20). The intention-to-treat analysis by cause using InterVA suggested fewer HIV/AIDS deaths in azithromycin-treated communities (rate ratio 0.70 [95% CI: 0.50–0.97]; *P* = 0.03) and fewer pneumonia deaths (rate ratio 0.82 [95% CI: 0.60–1.12]; *P* = 0.22). The use of the SmartVA algorithm suggested fewer diarrhea deaths (rate ratio 0.71 [95% CI: 0.51–1.00]; *P* = 0.05) and fewer pneumonia deaths (rate ratio 0.58 [95% CI: 0.33–1.00]; *P* = 0.05). Although this study is not able to provide strong evidence, the data suggest that the mortality reduction during MORDOR in Malawi may have been due to effects on pneumonia and diarrhea or HIV/AIDS mortality.

## INTRODUCTION

Mass drug administration (MDA) with azithromycin is widely used by trachoma control programs as part of efforts to eliminate trachoma, the leading infectious cause of blindness globally.^1^ During interventions principally aimed at eliminating trachoma, evidence emerged of reductions in a number of infectious diseases following azithromycin MDA, including diarrhea,^2^ pneumonia,^3^ and malaria.^4–6^ A study from Ethiopia reported a large reduction in child mortality following azithromycin MDA.^7^ Expert opinion suggested that a reduction in mortality was likely but to a lesser extent than estimated in this single available trial.^8^ MORDOR was a large multicenter trial that recently tested the hypothesis that biannual azithromycin MDA to children aged 1–59 months would reduce childhood mortality, reporting a 14% overall reduction in mortality in those assigned to azithromycin versus placebo.^9^ There is limited literature related to the mechanism through which azithromycin MDA may reduce child mortality. As a broad-spectrum antibiotic with weak antimalarial activity that has shown reductions in morbidity from pneumonia, diarrhea, and malaria, an effect on mortality from any of these major causes is feasible. In addition, azithromycin may reduce opportunistic infections in HIV-positive individuals, including *Mycobacterium avium* complex, and investigation is underway regarding its effect on HIV-associated chronic lung disease.^10,11^

Ideally, the underlying cause of death would be determined from medical certificates of cause of death completed by physicians with access to adequate investigative resources. However, at the MORDOR-Malawi study site, as in low-income settings in much of sub-Saharan Africa, there was limited access to physicians who would be able to complete death certificates. In such contexts, verbal autopsy (VA)—a structured interview with relatives of the deceased to ascertain cause of death—is increasingly used to infer likely causes of death. Development of standard electronic questionnaires on mobile devices and automated analysis programs have improved the feasibility of VA and comparability of results, compared with the previous requirement for physician review of the interviews.^12^ Prediction of cause-specific mortality fractions in a population using VA may be approximately 60–80% accurate, although lower for individual-level diagnosis.^13,14^ In settings where more accurate determination of cause of death is not possible, VA may be used to produce probable cause of death data with the caveat of it being an imperfect tool.^15^ The two most commonly used VA analysis programs are InterVA^16^ and SmartVA.^17^ InterVA uses a probabilistic model based on the relationship between indicators and causes, as captured through expert panel discussions.^18^ SmartVA, or the Tariff method, is based on the symptom–cause information from the Population Health Metrics Research Consortium (PHMRC) study that included more than 12,000 VA interviews performed on deaths with a gold standard diagnosis derived from stringent diagnostic criteria.^19,20^

The MORDOR trial was designed to assess overall mortality over three country sites and cause of death has not previously been compared between treatment arms for this study. Although azithromycin MDA has been shown to reduce morbidity due to the leading causes of child mortality outlined previously, there is no evidence to date for a cause-specific mortality effect of azithromycin MDA. As interest and evidence increases regarding the mortality benefit of azithromycin, an understanding of the mechanism for that benefit remains unclear. This study used VA methods to assess causes of death in children enrolled in the MORDOR trial in Malawi.

## METHODS

### Trial design and participants.

The methods for the main MORDOR trial in Niger, Tanzania, and Malawi are described elsewhere.^9^ Briefly, MORDOR assessed the effects of biannual single-dose azithromycin MDA compared with placebo on mortality in children aged 1–59 months. The trial was cluster randomized and in Malawi used the catchment area of a Health Surveillance Assistant (HSA) as a cluster. The study area covered the whole of Mangochi District and took place between March 2015 and June 2017, including a total of four treatment rounds and five census visits. Children were included if they were aged between 1 and 59 months at the start of any inter-census period. Biannual house-to-house census was conducted to identify all deaths as well as new births and migrations into or out of the study area.

The MORDOR trial included 304 clusters randomly selected from a pool of 334 clusters meeting the inclusion criteria identified from a pre-baseline census. The remaining 30 clusters, randomly located throughout the study area, were used for assessment of morbidity outcomes. This study, assessing cause-specific mortality, included deaths that occurred in all 334 communities, in the interests of presenting all available data.

### Interventions.

Azithromycin was administered at a dose of 20 mg/kg to all children available in study communities. Additional mop-up visits were conducted to increase the number of children treated. Children able to stand received an approximate dose based on their height, and small children were weighed. Placebo bottles and suspension were identical in appearance to azithromycin. Distribution of drug was performed by HSAs and MORDOR fieldworkers conducting house-to-house visits. Guardians were asked to inform the HSA of any adverse events that occurred within 7 days of receiving the study drug and HSAs were trained to inform the study team.

### Outcomes.

The primary prespecified outcome was cause of death, inferred using VA methods, for deaths in children aged 1–59 months at the prior census. A secondary prespecified outcome was seasonality of deaths. Both the InterVA and SmartVA automated analysis algorithms are endorsed by the WHO and performance overall is not greatly dissimilar between the methods.^14^ The sensitivity and specificity for diagnosis of specific causes does, however, vary considerably between the two algorithms, including for the four major causes of child mortality in this setting: malaria, pneumonia, diarrhea, and HIV/AIDS, as identified by Murray et al.^14^ and shown in Supplemental Table 7 for reference and discussion in the following paragraphs. This study used the WHO 2014 VA questionnaire with analysis using both the InterVA and SmartVA algorithms to provide a comprehensive and transparent assessment of the available data.

### Data collection.

Census data collection used custom-made software on Google Android devices. Deaths were identified from census updates and were followed up with VA interviews. Three nurses were trained to conduct VA interviews and aimed to complete these within 12 months of the death as recall is expected to diminish after this time.^21^ The WHO 2014 VA questionnaire was installed on Android devices using ODK Collect software. Data were uploaded to a secure server at the London School of Hygiene and Tropical Medicine.

### Sample size.

The MORDOR trial was designed to assess the effect of azithromycin on overall mortality in children aged 1–59 months with 84% power to detect a 15% effect over 2 years. The study was not designed to assess cause-specific mortality, which would require a significantly larger trial size.

### Randomization and blinding.

The study drug was labeled with 16 letters by the manufacturer (Pfizer Inc., New York, NY), with half corresponding to azithromycin and half to placebo. Communities, being the villages under a single HSA, were randomly assigned to a drug letter by the study statistician in San Francisco using the statistical package R (R Foundation for Statistical Computing, Vienna, Austria). All staff and participants in Malawi were blind to the treatment code until all data collection and cleaning was complete.

### Statistical methods.

The automated analysis programs used to produce inferred cause of death data, InterVA version 4 (InterVA version 4, University of Umeå, Umeå, Sweden) and SmartVA-Analyze (SmartVA-Analyze, Institute for Health Metrics and Evaluation, University of Washington, Seattle, WA), have thresholds for identifying the cause of death and if these are not met, the outcome is listed as “indeterminate” or “undetermined.”^16,17^ The SmartVA analysis software redistributes unknown causes of death according to the certainty of the algorithm in predicting different causes of death and Global Burden of Disease patterns for the country.^22^ As the SmartVA redistribution reflects the inherent ability of the program to predict each cause, redistribution may provide more accurate estimation of cause-specific mortality rates than the SmartVA output without redistribution; where possible, output with and without redistribution is presented. Output with redistribution is presented without CIs as this is produced by the SmartVA software at the population level rather than subsequently calculated from individual-level cause of death data.

Statistical analysis was performed using Stata version 15.1. Primary analyses were by intention-to-treat (ITT); to investigate if any cause-specific effects on mortality were due to individual or community-level effects of azithromycin, secondary per protocol (PP) analyses were performed that included only individuals who received treatment as indicated at the previous visit. Cause-specific mortality rates were calculated, and Poisson regression models produced, for the four main causes of mortality in the study area (pneumonia, diarrhea, HIV/AIDS, and malaria), with treatment allocation as the main predictor variable and random effects for clustering at the level of the MORDOR randomization unit. Point estimates with CIs from the models are presented as the best available estimates of the effect of azithromycin on cause-specific mortality in this population.

As an independent analysis to the VA algorithm outputs, the proportion of individuals with open response terms and positive responses to VA questions related to the major causes of child mortality were also assessed, specifically the open response terms “malaria,” “pneumonia,” and “diarrhea” and VA items “maternal test positive for HIV,” “frequent loose/liquid stool continuing until death,” “very severe cough,” and “severe fever”. A two-tailed test of proportions was used and *P* values are presented. Maternal HIV status was selected to estimate HIV exposure as very few children had a known HIV status, whereas mothers were more likely to have been tested for HIV during antenatal visits.

Study visits took place around the beginning and end of the dry season in Malawi, which occurs approximately between May and October. The baseline, 12-month, and 24-month visits took place between April and June 2015, 2016, and 2017, respectively; and the 6-month and 18-month visits between September and December 2015 and 2016. For the analysis of seasonality, dry season deaths were defined as those occurring between the baseline and 6-month visits and between the 12-month and 18-month visits, and wet season deaths as those occurring between the 6-month and 12-month visits and between the 18-month and 24-month visits. Univariate Poisson regression models were produced to assess the effects of treatment allocation during both the wet and dry seasons on each of the main causes of child mortality.

## RESULTS

In the 334 randomization units in the MORDOR trial in Malawi, there were 133,772 person-years of follow-up for children aged 1–59 months at the prior census. Person-years of follow-up and mortality rates were similar between males and females at each round, shown in Table 1. Over the course of the study, there were 1,184 deaths and VAs were completed for 1,131 of these. Median delay from death to VA was 5 months (range 1–26 months), with 87% of VAs completed within 12 months of death.

**Table 1:** Person-years enrolled in the study and number of deaths by follow-up period and gender

	Total person-years	Number of deaths (followed up with verbal autopsy)	Rate per 1,000 person-years (95% CI)
0–6 months follow-up			
Female	13,371	124 (118)	9.27 (7.78–11.06)
Male	13,398	135 (126)	10.08 (8.51–11.93)
Total	26,768	259 (244)	9.68 (8.57–10.93)
6–12 months follow-up			
Female	19,095	174 (168)	9.11 (7.85–10.57)
Male	19,031	181 (175)	9.51 (8.22–11.00)
Total	38,126	355 (343)	9.31 (8.39–10.33)
12–18 months follow-up			
Female	15,547	124 (123)	7.98 (6.69–9.51)
Male	15,356	117 (108)	7.62 (6.36–9.13)
Total	30,903	241 (231)	7.80 (6.87–8.85)
18–24 months follow-up			
Female	19,100	162 (154)	8.48 (7.27–9.89)
Male	18,875	167 (159)	8.85 (7.60–10.30)
Total	37,975	329 (313)	8.66 (7.78–9.65)
Grand total	133,772	1,184 (1,131)	8.85 (8.36–9.37)

Mortality was 9% lower in azithromycin-treated communities compared with those allocated to placebo (rate ratio 0.91 [95% CI: 0.79–1.05]; *P* = 0.20). The top 10 causes of death using the InterVA algorithm are shown in Table 2, and using the SmartVA algorithm in Table 3. Malaria was the leading cause of death overall, followed by HIV/AIDS, using both algorithms without redistribution of undetermined cases, and diarrhea, pneumonia, and either acute abdomen (InterVA) or other digestive diseases (SmartVA) made up the top five causes. The top 10 causes including redistribution of undetermined cases for SmartVA are also shown in Table 3. Pneumonia, malaria, diarrhea, and HIV/AIDS accounted for 83% and 78% of the inferred causes of death using InterVA and SmartVA with redistribution, respectively.

**Table 2:** Deaths in placebo- and azithromycin-treated clusters due to the top 10 causes using InterVA

InterVA output cause	Number of deaths in placebo arm (%)	Number of deaths in azithromycin arm (%)	Number of deaths in both arms (%)
Malaria	256 (42.9)	245 (45.9)	501 (44.3)
HIV/AIDS-related death	103 (17.3)	71 (13.3)	174 (15.4)
Acute respiratory infection, including pneumonia	94 (15.7)	77 (14.4)	171 (15.1)
Diarrheal diseases	48 (8.0)	45 (8.4)	93 (8.2)
Indeterminate	19 (3.2)	15 (2.8)	34 (3.0)
Acute abdomen	16 (2.7)	15 (2.8)	31 (2.7)
Meningitis and encephalitis	10 (1.7)	16 (3.0)	26 (2.3)
Severe malnutrition	12 (2.0)	9 (1.7)	21 (1.9)
Accidental exposure to smoke fire and flame	7 (1.2)	6 (1.1)	13 (1.2)
Epilepsy	2 (0.3)	9 (1.7)	11 (1.0)
Other	30 (5.0)	26 (4.9)	56 (4.9)
Total	597 (100)	534 (100)	1,131 (100)

**Table 3:** Deaths in placebo and azithromycin-treated clusters due to the top 10 causes using SmartVA

SmartVA output cause	Number of deaths in placebo arm (%)	Number of deaths in azithromycin arm (%)	Number of deaths in both arms (%)	Percentage of deaths in placebo arm after redistribution	Percentage of deaths in azithromycin arm after redistribution	Percentage of deaths in both arms after redistribution
Malaria	198 (33.2)	184 (34.5)	382 (33.8)	39.1	40.9	40.0
Undetermined	133 (22.3)	131 (24.5)	264 (23.3)	0.0	0.0	0.0
AIDS	71 (11.9)	70 (13.1)	141 (12.5)	12.8	13.2	13.0
Diarrhea/dysentery	79 (13.2)	56 (10.5)	135 (11.9)	16.1	13.0	14.6
Other digestive diseases	44 (7.4)	50 (9.4)	94 (8.3)	7.6	9.5	8.5
Pneumonia	35 (5.9)	20 (3.8)	55 (4.9)	10.3	10.0	10.1
Fires	8 (1.3)	6 (1.1)	14 (1.2)	1.5	1.3	1.4
Childhood cardiovascular diseases	5 (0.8)	4 (0.8)	9 (0.8)	1.1	1.1	1.1
Meningitis	5 (0.8)	3 (0.6)	8 (0.7)	2.0	2.5	2.2
Other infectious diseases	6 (1.0)	1 (0.2)	7 (0.6)	1.8	1.3	1.6
Other	13 (2.2)	9 (1.7)	22 (2.0)	7.7	7.2	7.5
Total	597 (100)	534 (100)	1,131 (100)	100	100	100

The ITT analyses of cause-specific mortality rates are shown using InterVA in Table 4 and SmartVA in Table 5. The analysis using InterVA showed 30% lower HIV/AIDS mortality in azithromycin-treated communities (rate ratio 0.70 [95% CI: 0.50–0.97]; *P* = 0.03), but this trend was not evident using SmartVA (rate ratio 0.99 [95% CI: 0.71–1.38]; *P* = 0.95) without redistribution and rate ratio 0.92 with redistribution. The effect estimate for pneumonia mortality was lower in azithromycin communities using both InterVA (rate ratio 0.82 [95% CI: 0.60–1.12]; *P* = 0.22) and SmartVA (rate ratio 0.58 [95% CI: 0.33–1.00]; *P* = 0.05) without redistribution and rate ratio 0.87 with redistribution.

**Table 4:** Cause-specific mortality by intention-to-treat for the four main inferred causes of death in the study area using InterVA

	Deaths/person-years	Rate per 1,000 person-years (95% CI)	Rate ratio* (95% CI)	*P*-value
Pneumonia				
Placebo	94/66,935	1.40 (1.15–1.72)	1	
Azithromycin	77/66,837	1.15 (0.92–1.44)	0.82 (0.60–1.12)	0.22
Malaria				
Placebo	256/66,935	3.82 (3.38–4.32)	1	
Azithromycin	245/66,837	3.67 (3.23–4.15)	0.95 (0.78–1.16)	0.64
HIV/AIDS				
Placebo	103/66,935	1.54 (1.27–1.87)	1	
Azithromycin	71/66,837	1.06 (0.84–1.34)	0.70 (0.50–0.97)	0.03
Diarrhea				
Placebo	48/66,935	0.72 (0.54–0.95)	1	
Azithromycin	45/66,837	0.67 (0.50–0.90)	0.95 (0.61–1.49)	0.84

* From random-effects Poisson model adjusting for clustering at the level of the randomization unit.

**Table 5:** Cause-specific mortality by intention-to-treat for the four main causes of death in the study area using SmartVA

	Number of cases/person-years	Rate per 1,000 person-years (95% CI)	Rate ratio* (95% CI)	*P*-value	Rate per 1,000 person-years after redistribution	Rate ratio after redistribution
Pneumonia						
Placebo	35/66,935	0.52 (0.38–0.73)	1		0.92	1
Azithromycin	20/66,837	0.30 (0.19–0.46)	0.58 (0.33–1.00)	0.05	0.80	0.87
Malaria						
Placebo	198/66,935	2.96 (2.57–3.40)	1		3.49	1
Azithromycin	184/66,837	2.75 (2.38–3.18)	0.93 (0.76–1.14)	0.49	3.27	0.94
HIV/AIDS						
Placebo	71/66,935	1.06 (0.84–1.34)	1		1.14	1
Azithromycin	70/66,837	1.05 (0.83–1.32)	0.99 (0.71–1.38)	0.95	1.05	0.92
Diarrhea						
Placebo	79/66,935	1.18 (0.95–1.47)	1		1.44	1
Azithromycin	56/66,837	0.84 (0.64–1.09)	0.71 (0.51–1.00)	0.05	1.04	0.72

* From random-effects Poisson model adjusting for clustering at the level of the randomization unit.

Inferred malaria mortality was similar in azithromycin communities compared with placebo using InterVA (rate ratio 0.95 [95% CI: 0.78–1.16]; *P* = 0.64) and SmartVA (rate ratio 0.93 [95% CI: 0.76–1.14]; *P* = 0.49) without redistribution and rate ratio 0.94 with redistribution. The effect estimate for diarrhea mortality was similar in azithromycin communities compared with placebo when analyzed using InterVA (rate ratio 0.95 [95% CI: 0.61–1.49]; *P* = 0.84) but was lower in azithromycin communities when analyzed using SmartVA (rate ratio 0.71 [95% CI: 0.51–1.00]; *P* = 0.05) without redistribution and rate ratio 0.72 with redistribution. The analyses of cause-specific mortality rates PP are shown in Supplemental Table 1 using InterVA, and Supplemental Table 2 using SmartVA. Cause-specific rate ratios PP were similar to those by ITT; within 10% for all causes.

**Figure 1.**
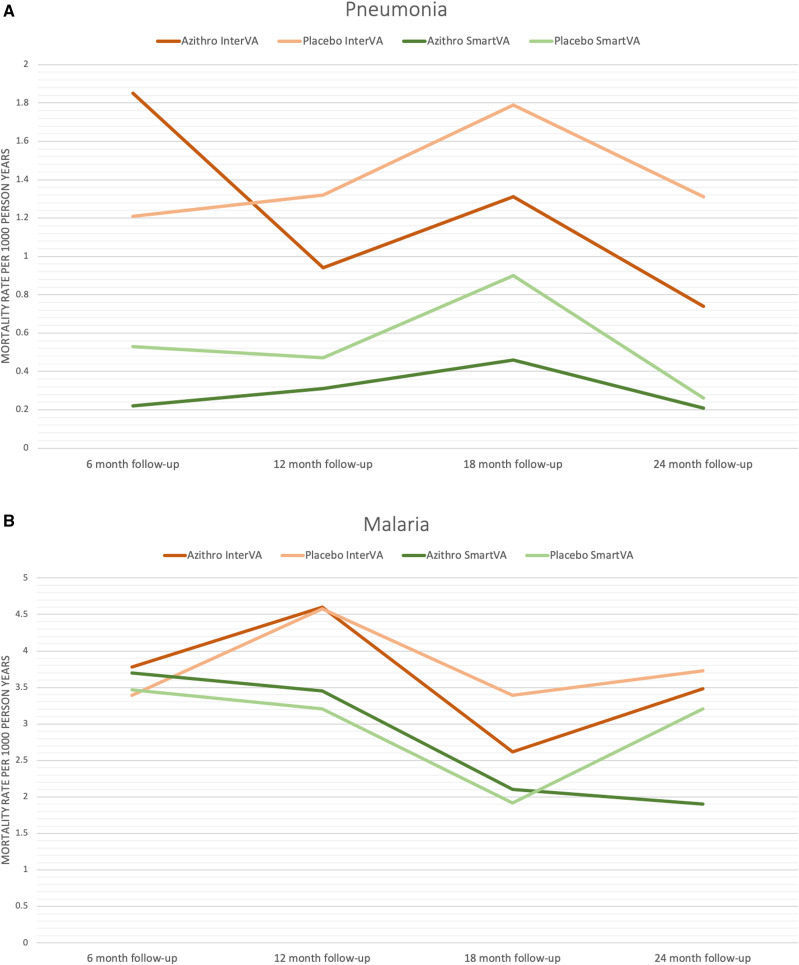

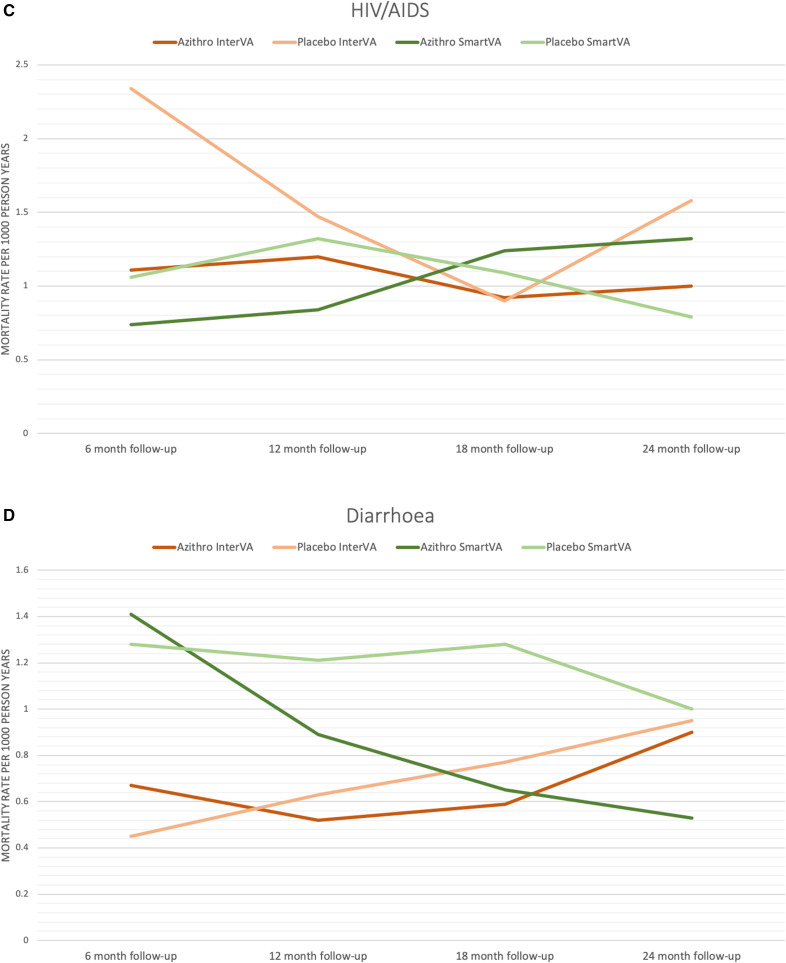
Cause-specific mortality rates by intention-to-treat for the leading causes of child mortality over the four follow-up periods of the study.

Cause-specific mortality rates for the leading causes of child mortality are shown by follow-up period in Figure 1. Pneumonia mortality rates were lower in azithromycin than placebo communities at three of the four follow-up visits when analyzed using InterVA and at all follow-up visits using SmartVA. HIV/AIDS mortality appears generally lower in azithromycin communities using InterVA and diarrhea mortality generally lower using SmartVA; no such patterns are evident for malaria mortality.

The comparison of VA response terms is shown in Table 6. In the ITT analysis, the open response term “pneumonia” was more frequently reported in VAs conducted in placebo communities than azithromycin: 8.2% versus 5.1%, respectively; *P* = 0.03. The open response term “diarrhea” was also more frequently reported in placebo than azithromycin communities: 21.9% versus 16.9%, respectively; *P* = 0.03. The VA item “frequent loose/liquid stool continuing until death” was more frequently endorsed in placebo than azithromycin communities: 37.2% versus 30.1%, respectively; *P* = 0.01. The open response term “malaria” was similar between placebo and azithromycin arms: 45.7% versus 49.1%, respectively; *P* = 0.26. “Maternal test positive for HIV,” “very severe cough,” and “severe fever” were all similar between treatment arms. The PP analyses showed similar trends to the ITT results.

**Table 6:** Comparison of verbal autopsy open response terms and question endorsements related to the major causes of child mortality between azithromycin- and placebo-treated communities

VA item	Intention-to-treat analysis			Per protocol analysis		
	Total deaths	Number of interviews in which item endorsed (%)	*P*-value*	Total deaths	Number of interviews in which item endorsed (%)	*P*-value*
Open response term “malaria”						
Placebo	597	273 (45.7)		461	220 (47.7)	
Azithromycin	534	262 (49.1)	0.26	431	217 (50.3)	0.44
Open response term “pneumonia”						
Placebo	597	49 (8.2)		461	40 (8.7)	
Azithromycin	534	27 (5.1)	0.03	431	22 (5.1)	0.04
Open response term “diarrhea”						
Placebo	597	131 (21.9)		461	96 (20.8)	
Azithromycin	534	90 (16.9)	0.03	431	75 (17.4)	0.19
Maternal test positive for HIV						
Placebo	597	45 (7.5)		461	35 (7.6)	
Azithromycin	534	41 (7.7)	0.93	431	37 (8.6)	0.59
Frequent loose/liquid stool continuing until death						
Placebo	597	222 (37.2)		461	170 (36.9)	
Azithromycin	534	161 (30.1)	0.01	431	133 (30.9)	0.06
Very severe cough						
Placebo	597	56 (9.4)		461	44 (9.5)	
Azithromycin	534	48 (9.0)	0.82	431	38 (8.8)	0.70
Severe fever						
Placebo	597	282 (47.2)		461	225 (48.8)	
Azithromycin	534	261 (48.9)	0.58	431	202 (46.9)	0.56

* From test of proportions.

Assessment of seasonality by ITT using InterVA and SmartVA did not identify clear trends for differences in cause-specific mortality between treatment arms; these analyses are included as Supplemental Data. Analysis using the InterVA algorithm suggested an effect of azithromycin on pneumonia mortality during the wet season but not the dry (wet season rate ratio 0.64 [95% CI: 0.41–1.00]; *P* = 0.05) and dry season rate ratio 1.02 [95% CI: 0.65–1.61]; *P* = 0.93) (Supplemental Table 3). Analysis using SmartVA, however, predicted approximately two-thirds fewer pneumonia deaths overall and the same seasonal trend was not evident (wet season rate ratio 0.71 [95% CI: 0.32–1.61]; *P* = 0.42 and dry season rate ratio 0.48 [95% CI: 0.23–1.02]; *P* = 0.06) (Supplemental Table 4). Analysis using InterVA suggested lower HIV/AIDS mortality in azithromycin communities during both the wet and dry seasons, whereas the SmartVA analysis suggested lower diarrhea mortality in azithromycin communities during both seasons. The PP analyses using InterVA and SmartVA show generally similar results to the ITT analysis and are included for completeness as Supplemental Tables 5 and 6, respectively.

## DISCUSSION

This study investigated the effect of azithromycin MDA on each of the major causes of child mortality in Mangochi District, Malawi, during the MORDOR trial. Verbal autopsy was used to infer the likely causes of death. The relative effects of azithromycin on cause-specific mortality during the wet and dry seasons was also assessed. The effect estimates produced in this study are mostly compatible with there being zero effect but are the only estimates available to date for the effect of azithromycin MDA on cause-specific child mortality.

The effect estimates using the InterVA analysis algorithm suggest lower pneumonia and HIV/AIDS mortality in azithromycin-treated than in placebo communities. Using SmartVA, the results suggest lower pneumonia and diarrhea mortality in azithromycin communities. The analysis of VA responses indicates that the terms “pneumonia” and “diarrhea” in the open narrative and “frequent loose/liquid stool continuing until death” were less commonly reported following deaths in azithromycin-treated than placebo communities. The relatively low sensitivity of InterVA for predicting both HIV/AIDS and diarrhea reported by Murray et al.,^14,19^ and shown in Supplemental Table 7, indicates the algorithm may underestimate mortality from these causes. Compared with SmartVA, InterVA did predict fewer diarrhea deaths, although more HIV/AIDS deaths in this study. The relatively higher sensitivity and lower specificity of InterVA for identifying pneumonia may lead to an overestimate of pneumonia mortality. SmartVA, on the other hand, has a particularly low sensitivity for pneumonia, which would contribute to the lower proportions of pneumonia reported in this study using the SmartVA algorithm, especially before redistribution of unknown causes.

The differences in sensitivity and specificity of the two algorithms reflect the fact that VA is a blunt diagnostic tool, less suited to individual-level diagnosis compared with population-level prediction of cause-specific mortality fractions. Nonetheless, primary analyses by the two algorithms are not necessarily contradictory, both suggesting lower pneumonia mortality in azithromycin communities and InterVA suggesting lower HIV/AIDS mortality, whereas SmartVA suggests lower diarrhea mortality. These diagnoses are particularly difficult to distinguish retrospectively in the absence of clinical investigation; diarrhea may often be the immediate cause of death when the underlying cause is HIV/AIDS. The direct analysis of VA responses would suggest lower pneumonia and diarrhea in the azithromycin group; the similar maternal HIV positivity between groups, estimating HIV exposure, does not provide evidence for whether or not the diarrhea is likely to be HIV-related. The PP analyses did not show any greater effect than the ITT analyses for pneumonia, diarrhea, or HIV/AIDS, providing no evidence that these were solely individual-level effects in those treated, as opposed to community-level effects.

An effect of azithromycin on respiratory and gastrointestinal infections, including on a background of HIV exposure or infection, leading to a reduction in the rate of pneumonia and diarrhea as immediate causes of death, is certainly plausible. Azithromycin is an effective treatment for community-acquired pneumonia, including the major etiological causes, *Streptococcus pneumoniae* and *Haemophilus influenzae*.^23^ Single-dose azithromycin has been shown to reduce oropharyngeal carriage of *S. pneumoniae* and decrease incidence of pneumonia.^24,25^

Azithromycin has efficacy against the main bacterial causes of fatal childhood diarrhea, namely, enteropathogenic and enterotoxigenic *Escherichia coli*, *Shigella* spp., *Campylobacter* spp., *Salmonella* spp., and *Vibrio cholerae*.^26^ Furthermore, azithromycin has anti-inflammatory properties that, combined with a reduction in gut pathogens, may reduce chronic immunostimulation caused by environmental enteric dysfunction and consequently improve nutritional status.^27,28^ Evidence from the MORDOR Niger study site indicates there may be changes to the gut microbiome following azithromycin MDA, although improvements in anthropometry measurements, as an assessment of nutritional status, have not previously been associated with azithromycin MDA.^29–31^

The REALITY trial identified a 27% reduction in mortality over 24 weeks in adults and children aged 5 years and older when commencing antiretroviral therapy (ART) plus an enhanced antimicrobial prophylactic regimen of trimethoprim–sulfamethoxazole, isoniazid–pyridoxine, fluconazole, albendazole, and a 5-day course of azithromycin, compared with commencing ART plus standard prophylaxis of trimethoprim–sulfamethoxazole only.^32^ The multiple dimensions to the enhanced regimen make it difficult to identify the specific intervention causing the reduction in mortality, but further analysis by Post et al.^33^ indicated mortality reductions from cryptococcosis and unknown causes, and not from severe bacterial infections, potentially azithromycin-responsive infections or tuberculosis.

REALITY investigated mortality in adults and older children during ART commencement, so it is not directly comparable with child mortality in the MORDOR trial; antimicrobial benefits and prophylactic effects of azithromycin against chronic disease-causing pathogens such as *M. avium* complex and *Pneumocystis jirovecii* could play a role in reducing HIV/AIDS mortality in low-resource settings where HIV is underdiagnosed.^34^ Prevalence of HIV infection in Malawi is approximately 9.2% in adults aged 15–49 years.^35^ HIV transmission is known to be a problem in fishing communities in Mangochi District because of practices associated with the market chain.^36^ Among HIV-infected children aged 0–14 years nationally, approximately 61% were estimated to be receiving ART in 2018.^35^ In the setting of significant levels of undiagnosed HIV/AIDS, an antibacterial agent could prevent or delay serious infections.

In this study, malaria was the most common cause predicted by both VA analysis algorithms but there was no evidence for an effect of azithromycin MDA on malaria mortality. Azithromycin is a weak antimalarial agent, and previous studies have indicated there may be reductions in malaria morbidity following azithromycin MDA.^4–6,37^ Recent assessment from the MORDOR trial in Niger indicated reduced malaria parasitemia in azithromycin-treated communities.^38^ However, morbidity assessments as part of the MORDOR trial in Tanzania indicated no difference in fever or anemia between azithromycin and placebo arms.^39^ A study comparing seasonal malaria chemoprevention (SMC) to SMC plus azithromycin in Burkina Faso and Mali, which have lower year-round malaria transmission than Malawi, reported no difference in mortality between groups, although reductions were evident in morbidity due to gastrointestinal and respiratory infections and nonmalarial febrile illness with the addition of azithromycin.^40^

A significant limitation of this study is that MORDOR was powered to detect a difference in overall mortality at three study sites and consequently the sample size is low for this study, first because it is limited to one of the three MORDOR sites and second because it is assessing cause-specific mortality rather than all-cause mortality. The study is underpowered to provide strong conclusions regarding cause-specific mortality rates, and the small effect estimates may represent type II error. Relatively small effect sizes for the major causes of child mortality may be clinically important and this study provides first estimates for how azithromycin may reduce child mortality for hypothesis generation and further investigation.

Verbal autopsy is recognized as the only feasible method for determining the cause of death in the absence of a clinical diagnosis in low-resource settings.^41^ Automated analysis of VA interviews improves the affordability and reproducibility of VA compared with physician review; however, the accuracy of individual-level diagnoses should not be assumed to be as accurate as medical certification of cause of death.^12^ Using the two main VA questionnaires, there is variation between the prediction of the main causes of death, so for the purposes of this investigation of the potential mechanisms of action of azithromycin, output from both algorithms was presented. The use of medical records and medical certificates of cause of death for in-facility deaths was investigated before the study but not pursued as, paradoxically, deaths that occurred in or on the way to a health facility were among the hardest to identify and categorize as there was no requirement for HSAs to report them, and health center data were generally absent or impossible to link to the community census. Any medical records retained by the family were reviewed by the VA team, and there are specific VA questions concerning diagnoses from the health system that are used in the algorithms for predicting cause of death.

In addition, VA, despite being used increasingly to estimate cause of death patterns where more sophisticated methods are not practicable, is a relatively imprecise instrument that relies on details of the final illness recalled by family members. Accuracy of recall for VA has been estimated to decrease by approximately 0.6% per month following the death, and it is recommended that, where possible, VA should be completed within 12 months.^21,42^ Verbal autopsy completion for this study took place as soon as possible following identification of the death from the census, and after the customary 1-month mourning period, however, many visits were required to trace family members for some children as households in the study area move quite frequently, especially after a child’s death. The median delay of 5 months in this study and with 87% of VAs completed within 12 months is within the normally accepted range and is not expected to have significantly affected the accuracy of predicted diagnoses.

Finally, this study used the WHO 2014 VA questionnaire, which was designed to facilitate the use of both SmartVA and InterVA for assigning cause of death.^43^ The 2014 instrument includes all questions required for InterVA, although not the exact wording for all questions from the PHMRC questionnaire used by SmartVA. A further updated questionnaire, released in 2016 after this study started, is fully compatible with both algorithms. Most questions required as input for SmartVA are similar to those in the 2014 questionnaire, although the performance of SmartVA may be slightly reduced compared with having input from the WHO 2016 questionnaire or PHMRC questionnaire. The benefits of having analysis from these two main algorithms for a wider discussion of the implications of the data were deemed to outweigh the potential for a slight decrease in performance. There is a more recently developed statistical tool for producing cause of death data from VA interviews (InSilicoVA), which aimed to improve on the other methods by sharing uncertainty between cause of death assigned for specific individuals and the population distribution of causes of death.^44^ Adding a third analysis method with associated increased complexity was not attempted for this study.

Although this study is not able to provide strong evidence on the causes of death in the MORDOR trial, the data have been presented fully to enable generation of hypotheses regarding mechanisms of effect of azithromycin on child mortality. The data suggest the mortality reduction in the MORDOR trial in Malawi may have been due to effects on pneumonia and diarrhea or HIV/AIDS mortality. Larger studies will be required to clearly define the effects of azithromycin MDA on cause-specific child mortality.

## Supplemental tables

Supplemental materials
