# Supplementary material for: Effect of Mass Treatment with Azithromycin on Causes of Death in Children in Malawi: Secondary Analysis from the MORDOR Trial
Source: Am J Trop Med Hyg. 2020 Apr 27;103(3):1319–28. doi: 10.4269/ajtmh.19-0613 (PMC7470551; doi:10.4269/ajtmh.19-0613)
Supplement: Supplementary file 1 [file tpmd190613.SD1.docx]

**Supplemental Table 1:** Cause-specific mortality per-protocol for the four main causes of death in the study area *using InterVA*

|  | **Number of cases/person-years** | **Rate per 1000 person-years (95% CI)** | **Rate Ratio***  **(95% CI)** | ***P-*value** |
| --- | --- | --- | --- | --- |
| **Pneumonia** |  |  |  |  |
| Placebo | 71/58,832 | 1.21 (0.96-1.52) | 1 |  |
| Azithro | 62/58,731 | 1.06 (0.82-1.35) | 0.88 (0.62-1.24) | 0.46 |
| **Malaria** |  |  |  |  |
| Placebo | 204/58,832 | 3.47 (3.02-3.98) | 1 |  |
| Azithro | 202/58,731 | 3.44 (3.00-3.95) | 0.99 (0.80-1.23) | 0.92 |
| **HIV/AIDS** |  |  |  |  |
| Placebo | 71/58,832 | 1.21 (0.96-1.52) | 1 |  |
| Azithro | 56/58,731 | 0.95 (0.73-1.24) | 0.80 (0.54-1.19) | 0.27 |
| **Diarrhea** |  |  |  |  |
| Placebo | 37/58,832 | 0.63 (0.46-0.87) | 1 |  |
| Azithro | 38/58,731 | 0.65 (0.47-0.89) | 1.05 (0.64-1.72) | 0.85 |

**From random effects Poisson model adjusting for clustering at the level of the randomization unit*

**Supplemental Table 2:** Cause-specific mortality per-protocol for the four main causes of death in the study area *using SmartVA*

|  | **Number of cases/person-years** | **Rate per 1,000 person-years (95% CI)** | **Rate Ratio***  **(95% CI)** | ***P-*value** | **Rate after redistribution** | **Rate ratio after redistribution** |
| --- | --- | --- | --- | --- | --- | --- |
| **Pneumonia** |  |  |  |  |  |  |
| Placebo | 24/58,832 | 0.41 (0.27-0.61) | 1 |  | 0.74 | 1 |
| Azithro | 16/58,731 | 0.27 (0.17-0.44) | 0.67 (0.36-1.27) | 0.22 | 0.71 | 0.96 |
| **Malaria** |  |  |  |  |  |  |
| Placebo | 154/58,832 | 2.62 (2.24-3.07) | 1 |  | 3.10 | 1 |
| Azithro | 154/58,731 | 2.62 (2.24-3.07) | 1.00 (0.80-1.25) | 0.98 | 3.01 | 0.97 |
| **HIV/AIDS** |  |  |  |  |  |  |
| Placebo | 58/58,832 | 0.99 (0.76-1.28) | 1 |  | 1.05 | 1 |
| Azithro | 62/58,731 | 1.06 (0.82-1.35) | 1.07 (0.75-1.53) | 0.70 | 1.06 | 1.01 |
| **Diarrhea** |  |  |  |  |  |  |
| Placebo | 57/58,832 | 0.97 (0.75-1.26) | 1 |  | 1.18 | 1 |
| Azithro | 46/58,731 | 0.78 (0.59-1.05) | 0.81 (0.55-1.20) | 0.29 | 0.97 | 0.82 |

**From random effects Poisson model adjusting for clustering at the level of the randomization unit*

**Supplemental Table 3:** Seasonality of cause-specific mortality by intention-to-treat *using InterVA*

|  | **Wet season** | | | | **Dry season** | | | |
| --- | --- | --- | --- | --- | --- | --- | --- | --- |
|  | **Deaths /person-years** | **Rate per 1000 person-years (95% CI)** | **Rate Ratio***  **(95% CI)** | ***P-*value** | **Deaths /person-years** | **Rate per 1000 person-years (95% CI)** | **Rate Ratio***  **(95% CI)** | ***P-*value** |
| **Pneumonia** |  |  |  |  |  |  |  |  |
| Placebo | 50/38,035 | 1.31 (1.00-1.73) | 1 |  | 44/28,900 | 1.52 (1.13-2.05) | 1 |  |
| Azithro | 32/38,066 | 0.84 (0.59-1.19) | 0.64 (0.41-1.00) | 0.05 | 45/28,772 | 1.56 (1.17-2.09) | 1.02 (0.65-1.61) | 0.93 |
| **Malaria** |  |  |  |  |  |  |  |  |
| Placebo | 158/38,035 | 4.15 (3.55-4.86) | 1 |  | 99/28,900 | 3.43 (2.81-4.17) | 1 |  |
| Azithro | 154/38,066 | 4.05 (3.45-4.74) | 0.97 (0.75-1.26) | 0.84 | 92/28,772 | 3.20 (2.61-3.92) | 0.92 (0.68 -1.26) | 0.62 |
| **HIV/AIDS** |  |  |  |  |  |  |  |  |
| Placebo | 58/38,035 | 1.52 (1.18-1.97) | 1 |  | 45/28,900 | 1.56 (1.16-2.09) | 1 |  |
| Azithro | 42/38,066 | 1.10 (0.82-1.49) | 0.72 (0.47-1.10) | 0.13 | 29/28,772 | 1.01 (0.70-1.45) | 0.67 (0.40-1.11) | 0.12 |
| **Diarrhoea** |  |  |  |  |  |  |  |  |
| Placebo | 30/38,035 | 0.79 (0.55-1.13) | 1 |  | 18/28,900 | 0.62 (0.39-0.99) | 1 |  |
| Azithro | 27/38,066 | 0.71 (0.49-1.03) | 0.92 (0.51-1.63) | 0.76 | 18/28,772 | 0.63 (0.39-0.99) | 1.02 (0.50-2.09) | 0.95 |

**From univariate Poisson regression*

**SupplementalTable 4:** Seasonality of cause-specific mortality by intention-to-treat *using SmartVA* (without redistribution of unknown causes of death)

|  | **Wet season** | | | | **Dry season** | | | |  |
| --- | --- | --- | --- | --- | --- | --- | --- | --- | --- |
|  | **Deaths /person-years** | **Rate per 1000 person-years (95% CI)** | **Rate Ratio***  **(95% CI)** | ***P-*value** | **Deaths /person-years** | **Rate per 1000 person-years (95% CI)** | **Rate Ratio***  **(95% CI)** | ***P-*value** | |
| **Pneumonia** |  |  |  |  |  |  |  |  | |
| Placebo | 14/38,035 | 0.37 (0.22-0.62) | 1 |  | 21/28,900 | 0.73 (0.47-1.11) | 1 |  | |
| Azithro | 10/38,066 | 0.26 (0.14-0.49) | 0.71 (0.32-1.61) | 0.42 | 10/28,772 | 0.35 (0.19-0.65) | 0.48 (0.23-1.02) | 0.06 | |
| **Malaria** |  |  |  |  |  |  |  |  | |
| Placebo | 122/38,035 | 3.21 (2.69-3.83) | 1 |  | 76/28,900 | 2.63 (2.10-3.29) | 1 |  | |
| Azithro | 102/38,066 | 2.68 (2.21-3.25) | 0.84 (0.64-1.09) | 0.18 | 82/28,772 | 2.85 (2.30-3.54) | 1.08 (0.79 -1.48) | 0.61 | |
| **HIV/AIDS** |  |  |  |  |  |  |  |  | |
| Placebo | 40/33,093 | 1.05 (0.77-1.43) | 1 |  | 31/25,739 | 1.07 (0.75-1.53) | 1 |  | |
| Azithro | 41/33,086 | 1.08 (0.79-1.46) | 1.02 (0.66-1.58) | 0.91 | 29/25,645 | 1.01 (0.70-1.45) | 0.94 (0.57-1.56) | 0.81 | |
| **Diarrhea** |  |  |  |  |  |  |  |  | |
| Placebo | 42/38,035 | 1.10 (0.82-1.49) | 1 |  | 37/28,900 | 1.28 (0.93-1.77) | 1 |  | |
| Azithro | 27/38,066 | 0.71 (0.49-1.03) | 0.64 (0.40-1.04) | 0.07 | 29/28,772 | 1.01 (0.70-1.45) | 0.79 (0.48-1.28) | 0.34 | |

**From univariate Poisson regression*

**Supplemental Table 5:** Seasonality of cause-specific mortality per-protocol *using InterVA*

|  | **Wet season** | | | | **Dry season** | | | |
| --- | --- | --- | --- | --- | --- | --- | --- | --- |
|  | **Deaths /person-years** | **Rate per 1000 person-years (95% CI)** | **Rate Ratio***  **(95% CI)** | ***P-*value** | **Deaths /person-years** | **Rate per 1000 person-years (95% CI)** | **Rate Ratio***  **(95% CI)** | ***P-*value** |
| **Pneumonia** |  |  |  |  |  |  |  |  |
| Placebo | 42/33,093 | 1.27 (0.94-1.72) | 1 |  | 29/25,739 | 1.13 (0.78-1.62) | 1 |  |
| Azithro | 26/33,086 | 0.79 (0.54-1.15) | 0.62 (0.38-1.01) | 0.06 | 36/25,645 | 1.40 (1.01-1.95) | 1.24 (0.75-2.06) | 0.40 |
| **Malaria** |  |  |  |  |  |  |  |  |
| Placebo | 133/33,093 | 4.02 (3.39-4.76) | 1 |  | 71/25,739 | 2.76 (2.19-3.48) | 1 |  |
| Azithro | 135/33,086 | 4.08 (3.45-4.83) | 1.01 (0.77-1.33) | 0.94 | 67/25,645 | 2.61 (2.06-3.32) | 0.94 (0.67-1.34) | 0.75 |
| **HIV/AIDS** |  |  |  |  |  |  |  |  |
| Placebo | 46/33,093 | 1.39 (1.04-1.86) | 1 |  | 25/25,739 | 0.97 (0.66-1.44) | 1 |  |
| Azithro | 36/33,086 | 1.09 (0.78-1.51) | 0.77 (0.48-1.24) | 0.29 | 20/25,645 | 0.78 (0.50-1.21) | 0.87 (0.44-1.69) | 0.67 |
| **Diarrhea** |  |  |  |  |  |  |  |  |
| Placebo | 23/33,093 | 0.70 (0.46-1.05) | 1 |  | 14/25,739 | 0.54 (0.32-0.92) | 1 |  |
| Azithro | 26/33,086 | 0.79 (0.54-1.15) | 1.16 (0.62-2.18) | 0.65 | 12/25,645 | 0.47 (0.27-0.82) | 0.87 (0.39-1.93) | 0.73 |

**From univariate Poisson regression*

**Supplemental Table 6:** Seasonality of cause-specific mortality per protocol *using SmartVA* (without redistribution of unknown causes of death)

|  | **Wet season** | | | | **Dry season** | | | |
| --- | --- | --- | --- | --- | --- | --- | --- | --- |
|  | **Deaths /person-years** | **Rate per 1000 person-years (95% CI)** | **Rate Ratio***  **(95% CI)** | ***P-*value** | **Deaths /person-years** | **Rate per 1000 person-years (95% CI)** | **Rate Ratio***  **(95% CI)** | ***P-*value** |
| **Pneumonia** |  |  |  |  |  |  |  |  |
| Placebo | 13/33,093 | 0.39 (0.23-0.68) | 1 |  | 11/25,739 | 0.43 (0.24-0.77) | 1 |  |
| Azithro | 8/33,086 | 0.24 (0.12-0.48) | 0.62 (0.26-1.49) | 0.28 | 8/25,645 | 0.31 (0.16-0.62) | 0.73 (0.29-1.81) | 0.50 |
| **Malaria** |  |  |  |  |  |  |  |  |
| Placebo | 98/33,093 | 2.96 (2.43-3.61) | 1 |  | 56/25,739 | 2.18 (1.67-2.83) | 1 |  |
| Azithro | 90/33,086 | 2.72 (2.21-3.34) | 0.92 (0.69-1.22) | 0.56 | 64/25,645 | 2.50 (1.95-3.19) | 1.15 (0.80-1.64) | 0.45 |
| **HIV/AIDS** |  |  |  |  |  |  |  |  |
| Placebo | 35/38,035 | 1.06 (0.76-1.47) | 1 |  | 23/28,900 | 0.89 (0.59-1.34) | 1 |  |
| Azithro | 38/38,066 | 1.15 (0.84-1.58) | 1.09 (0.69-1.72) | 0.73 | 24/28,772 | 0.94 (0.63-1.40) | 1.05 (0.59-1.89) | 0.87 |
| **Diarrhea** |  |  |  |  |  |  |  |  |
| Placebo | 33/33,093 | 1.00 (0.71-1.40) | 1 |  | 24/25,739 | 0.93 (0.62-1.39) | 1 |  |
| Azithro | 25/33,086 | 0.76 (0.51-1.12) | 0.76 (0.45-1.27) | 0.30 | 21/25,645 | 0.82 (0.53-1.26) | 0.88 (0.49-1.58) | 0.66 |

**From univariate Poisson regression*

**Supplemental Table 7:** Sensitivity and specificity of the VA algorithms for predicting the leading causes of child death

|  | **InterVA4** | | **Tariff 2.0** | |
| --- | --- | --- | --- | --- |
|  | **Sensitivity** | **Specificity** | **Sensitivity** | **Specificity** |
| Malaria | 44.7 | 89.2 | 59.3 | 92.8 |
| HIV | 28.2 | 95.5 | 60 | 96.9 |
| Pneumonia | 75 | 64.3 | 14.2 | 98.2 |
| Diarrhea | 28.4 | 98.1 | 40 | 95.5 |

Data from Murray et al., 2014
